# Unlocking glioma vulnerabilities: targeting regulated cell death pathways for innovative therapies

**DOI:** 10.1038/s41420-026-02949-8

**Published:** 2026-02-10

**Authors:** Jincai Guo, Lijuan Zong, Ying Huang, Xiang Liu, Yixiang Hu, Ya Liu

**Affiliations:** 1 Department of Pharmacy, Changsha Stomatological Hospital, Changsha, China; 2 Department of Rehabilitation Medicine, Zhongda Hospital of Southeast University, Nanjing, China; 3 Zhongshan Hospital of Traditional Chinese Medicine Afflilated to Guangzhou University of Chinese Medicine, Zhongshan, China; 4 Department of Clinical Pharmacy, The Central Hospital of Xiangtan (The affiliated hospital of Hunan University), Xiangtan, China

**Keywords:** CNS cancer, Cell death

## Abstract

Glioma, the most prevalent primary brain tumor, primarily arises from glial cells or their progenitors. Histologically, gliomas are classified into astrocytomas, oligodendrogliomas, and ependymomas. Due to their aggressive invasive nature and resistance to chemotherapy, gliomas exhibit high recurrence rates and poor clinical outcomes. Regulated cell death (RCD) refers to a set of genetically controlled cellular processes that significantly influence tumor behavior. RCD plays a dual role in cancer: under normal physiological conditions, it eliminates malignant cells to prevent tumorigenesis, while in pathological conditions, tumor cells evade RCD to gain survival advantages. Furthermore, distinct RCD pathways can modulate the tumor immune microenvironment, thereby affecting therapeutic outcomes. Targeting RCD mechanisms presents a promising strategy to overcome therapeutic resistance and advance innovative glioma immunotherapies. This review explores the molecular mechanisms of pyroptosis, ferroptosis, necroptosis, and autophagy in glioma, emphasizing their critical roles in tumor progression. It also examines therapeutic strategies targeting RCD, including recent advancements in glutathione peroxidase 4 (GPX4) inhibitors, oncolytic virotherapy, and other emerging agents. Furthermore, the review discusses the potential of nanoparticle-based drug delivery systems and multi-omics approaches to optimize personalized combination therapies, aiming to enhance multimodal, synergistic interventions for more effective glioma management.

## Facts

- Regulated cell death (RCD) pathways, including pyroptosis, ferroptosis, necroptosis, and autophagy, play critical roles in glioma progression and immune modulation.
- RCD mechanisms can shape the tumor microenvironment, affecting glioma progression and treatment response.
- Targeting RCD pathways presents a promising approach to overcoming glioma’s resistance to traditional therapies.
- A deeper understanding of how RCD impacts the tumor microenvironment could lead to novel strategies for enhancing anti-tumor immunotherapies.

## Open Questions

- How can gliomas be sensitized to RCD pathways to overcome treatment resistance?
- How can intratumoral heterogeneity and blood-brain barrier (BBB) permeability be addressed to improve RCD-targeted therapies for glioma?
- How do immune responses induced by RCD influence glioma progression, and can these responses be modulated to improve anti-tumor immunity?

## Introduction

Gliomas are the most prevalent primary brain tumors, accounting for approximately 70% of adult central nervous system neoplasms and representing the dominant class of brain malignancies in adults [1, 2]. These tumors typically originate from glial cells or their progenitors and develop into various subtypes, including astrocytomas, oligodendrogliomas, ependymomas, and oligoastrocytomas. According to the World Health Organization (WHO) classification, gliomas are stratified into four grades: grades 1 and 2 are classified as low-grade gliomas (LGGs), while grades 3 and 4 represent high-grade gliomas (HGGs). Epidemiological data from the Central Brain Tumor Registry of the United States (CBTRUS) report an age-adjusted annual incidence of approximately 5.95 cases per 100,000 individuals, with glioblastoma (GBM) being the most frequent and aggressive subtype, accounting for 3.23 cases per 100,000 individuals annually [3].

**Fig. 1:**
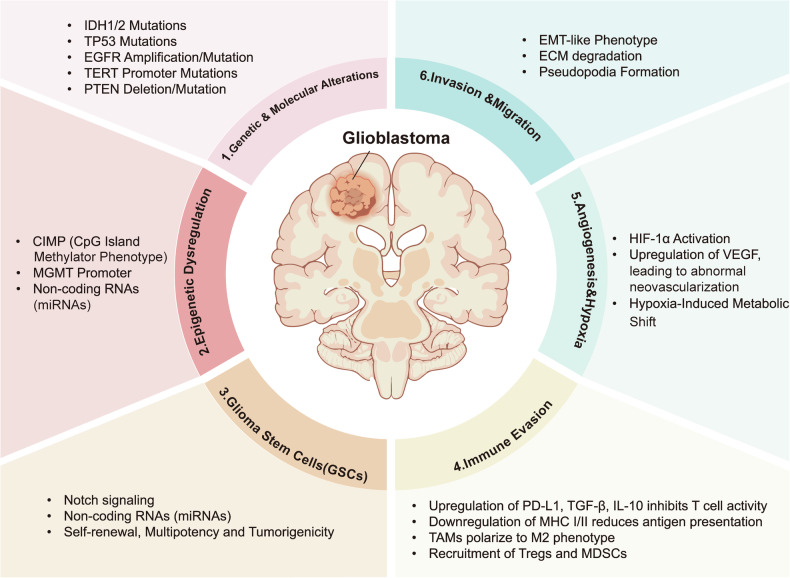
Six major pathological mechanisms contributing to glioblastoma (GBM) progression. (1) Genetic and Molecular Alterations, including IDH1/2 and TP53 mutations, EGFR amplification/mutation, TERT promoter mutations, and PTEN loss; (2) Epigenetic Dysregulation, such as CIMP status, MGMT promoter methylation, and dysregulated non-coding RNAs (e.g., miRNAs); (3) Glioma Stem Cells (GSCs), characterized by Notch pathway activation, miRNA-mediated regulation, and enhanced self-renewal and tumorigenicity; (4) Immune Evasion, involving PD-L1, TGF-β, and IL-10-mediated T cell inhibition, MHC downregulation, M2-type TAM polarization, and recruitment of Tregs/MDSCs; (5) Angiogenesis and Hypoxia, driven by HIF-1α and VEGF upregulation resulting in abnormal neovascularization and metabolic reprogramming; and (6) Invasion and Migration, featuring EMT-like transitions, extracellular matrix (ECM) degradation, and pseudopodia formation. These interrelated mechanisms collectively contribute to the therapeutic resistance and aggressive nature of GBM.

The pathogenesis of gliomas is multifactorial and involves complex interactions including genetic and molecular alterations (e.g., IDH1/2, TP53, and EGFR amplification), epigenetic alterations (e.g., MGMT promoter methylation), glioma stem cells (GSCs) dysfunction (e.g., Notch pathway activation enhances self-renewal and tumorigenicity), immune Evasion (e.g., PD-L1 and TGF-β-mediated T cell inhibition), angiogenesis and hypoxia (e.g., HIF-1α and VEGF upregulation promotes abnormal angiogenesis and metabolism), and invasion characterized by epithelial-mesenchymal transition (EMT)-like transitions (Fig. 1). Despite decades of research and therapeutic advances, only a limited number of glioma-targeted agents have been approved by the U.S. Food and Drug Administration (FDA). The poor clinical outcomes are largely attributed to the tumor’s intrinsic resistance to therapy, driven by factors such as blood-brain barrier (BBB) impermeability, cellular heterogeneity, and the persistence of glioma stem cells (GSCs). Consequently, conventional treatment modalities, including surgical resection, radiotherapy, and chemotherapy, often fail to prevent tumor recurrence [4].

Regulated cell death (RCD) refers to a set of genetically controlled cellular demise processes, including apoptosis, pyroptosis, ferroptosis, necroptosis, and autophagy [5]. In tumorigenesis, RCD plays a dual role: physiologically, it suppresses tumor initiation by eliminating damaged or mutated cells; however, in tumor microenvironment, cancer cells circumvent RCD through strategies such as upregulating anti-apoptotic proteins and enhancing antioxidant defenses, thereby promoting tumor survival and progression [6]. Furthermore, RCD is intricately linked to the TME. Different RCD modalities release pathogen-associated molecular patterns (PAMPs) and damage-associated molecular patterns (DAMPs), which modulate immune surveillance and influence antitumor immunity [7, 8]. These findings highlight RCD as a critical mechanism in therapeutic resistance and a key target for the development of novel immunotherapeutic strategies.

To inform this narrative review, we searched PubMed, EMBASE, and Web of Science for studies published between January 2010 and June 2025, using keywords related to glioma and regulated cell death pathways, including pyroptosis, ferroptosis, necroptosis, autophagy, and others. Studies were selected based on their relevance to glioma and key RCD pathways, focusing on preclinical and clinical research that highlights the dual roles of these pathways in glioma progression. We summarize recent advances in targeting RCD, including the use of glutathione peroxidase 4 (GPX4) inhibitors and oncolytic virotherapy to overcome resistance and activate immune responses. Emerging strategies, such as nanoparticle-based delivery systems and multi-omics-guided personalized therapies, offer spatiotemporal control of RCD induction while minimizing neurotoxicity. Finally, we explore the potential synergy between RCD modulation and immune checkpoint blockade, providing a framework for multimodal approaches to glioma treatment.

## Overview of RCD Mechanisms In Glioma

Conventional forms of cell death primarily refer to apoptosis and necrosis. Apoptosis, the earliest identified form of programmed cell death, is tightly regulated through two primary pathways: the intrinsic (mitochondrial) pathway—mediated by Bax/Bak-induced cytochrome *c* release—and the extrinsic (death receptor) pathway, which involves Fas or TRAIL-induced caspase-8 activation, leading to nuclear fragmentation and apoptotic body formation [9]. Gliomas frequently evade apoptosis by overexpressing anti-apoptotic proteins such as Bcl-2 or activating survival pathways like PI3K/Akt, thereby promoting cell survival and resistance to therapy. Traditionally regarded as a passive process, necrosis is now understood to be capable of active immunomodulatory functions. Necrotic cells can release DAMPs, such as high-mobility group box 1 (HMGB1), which activate the Toll-like receptor 4 (TLR4)/NF-κB signaling axis. This cascade triggers pro-inflammatory immune responses that may exacerbate inflammation within the TME and potentially promote tumor progression and metastasis.

**Fig. 2:**
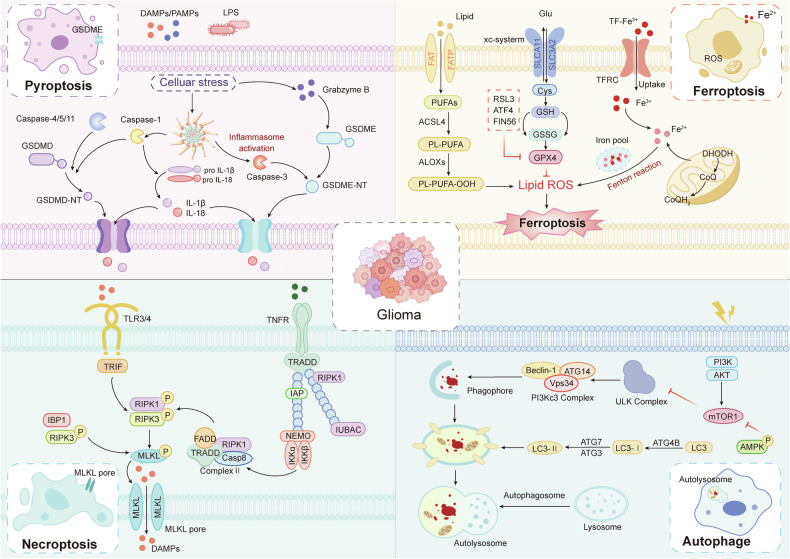
Pathways controlling pyroptosis, ferroptosis, necroptosis, and autophagy. Pyroptosis is a form of inflammatory RCD triggered by pathogen-associated molecular patterns (PAMPs), damage-associated molecular patterns (DAMPs), and lipopolysaccharide (LPS). It involves the activation of inflammasomes in a caspase-dependent manner and subsequent cleavage of gasdermin family proteins, leading to membrane pore formation, cell membrane rupture, and the release of pro-inflammatory cytokines, notably IL-1β and IL-18. Ferroptosis is initiated by the iron-dependent Fenton reaction, rendering its sensitivity tightly linked to iron metabolism. This process includes iron uptake (e.g., via transferrin), intracellular storage (e.g., via ferritin), mobilization (e.g., mediated by NCOA4), and export (e.g., via PROM2). Additionally, the ACSL4/LPCAT3/ALOX signaling axis, along with RAB7A-dependent lipophagy, accelerates ferroptosis through enhanced lipid peroxidation and subsequent phospholipid hydroperoxide (PLOOH) accumulation. Cellular defense against ferroptosis relies on both GPX4-dependent and GPX4-independent antioxidant systems that suppress lipid peroxidation. Necroptosis is activated via cell surface death receptors (such as Fas receptors, TNFRs, IFN receptors, and TLRs) and the intracellular sensor ZBP1. The execution of necroptosis is regulated by multiple critical checkpoints, including the post-translational modifications of RIPK1, the enzymatic activity of caspase-8, RHIM-dependent homodimerization of RIPK3, phosphorylation of MLKL, NINJ1-mediated disruption of the plasma membrane, and membrane repair mechanisms orchestrated by the ESCRT-III complex. Autophagy is a highly regulated self-degradation pathway activated by nutrient scarcity through mTOR signaling and energy depletion via AMPK activation, both leading to the stimulation of VPS34. The Beclin-1/VPS34 complex drives the initiation and expansion of autophagic membranes. Activation of JNK kinase phosphorylates BCL-2 and BIM, disrupting their inhibitory binding to Beclin-1 and thus freeing Beclin-1 to associate with VPS34 and promote PI3P production, essential for autophagosome elongation. LC3 lipidation is mediated by ubiquitin-like conjugation systems involving ATG7, ATG3, and the ATG5-ATG12-ATG16L complex, facilitating cargo recognition and sequestration. Ultimately, autophagosomes fuse with lysosomes to form autolysosomes where lysosomal hydrolases degrade the cargo, enabling the recycling of cellular nutrients.

In recent years, the identification of novel forms of RCD has broadened the therapeutic landscape for glioma (Fig. 2). Pyroptosis is a pro-inflammatory form of cell death mediated by the gasdermin (GSDM) family. It is typically initiated by the cleavage of GSDMD via inflammatory caspases (caspase-1/4/5/11) or GSDME by apoptotic caspase-3. The cleavage of N-terminal domain forms membrane pores, leading to cell lysis and the release of interleukin (IL)-1β and IL-18, which amplify immune responses [10]. In glioma, GSDME expression is often silenced by promoter methylation; however, reactivation of GSDME can induce chemotherapy-associated pyroptosis—such as that triggered by cisplatin—resulting in tumor antigen release and enhanced CD8⁺ T-cell infiltration. Furthermore, GSDME hypermethylation correlates with an immunosuppressive TME and serves as a prognostic biomarker [11, 12]. Ferroptosis is an iron-dependent form of regulated necrosis driven by lipid peroxidation. Key molecular regulators include GPX4, system Xc^−^ (cystine/glutamate antiporter), and acyl-CoA synthetase long-chain family member 4 (ACSL4). Ferroptosis can be induced via GPX4 inhibition (e.g., RSL3), system Xc⁻ blockade (e.g., erastin), or ACSL4-mediated polyunsaturated fatty acid (PUFA) peroxidation. Glioma cells exhibit high metabolic demand and are thus particularly vulnerable to ferroptosis; however, they often develop resistance through activation of the Nrf2 signaling pathway or upregulation of SLC7A11 [13, 14]. IDH1-mutant gliomas are notably sensitive to ferroptosis inducers such as sorafenib, potentially due to 2-hydroxyglutarate (2-HG)-mediated suppression of GPX4 [15]. Combining ferroptosis inducers with iron chelators (e.g., deferoxamine) can mitigate collateral damage to healthy brain tissue, while nanoparticle-based drug delivery (e.g., liposomal formulations) enhances BBB penetration [16]. Necroptosis is a lytic, immunogenic form of RCD executed through the RIPK1/RIPK3/MLKL signaling axis. In glioma, downregulation of RIPK3 contributes to immune evasion. Activation of necroptosis via RIPK3 agonists such as GSK872, particularly when combined with immune checkpoint inhibitors (e.g., PD-1 blockade), enhances tumor-associated macrophage (TAM) phagocytosis and reverses the immunologically “cold” tumor phenotype. Inhibition of the CD47-SIRPα signaling axis using agents like RRx-001 can further potentiate necroptosis by disabling the “don’t eat me” signal, thereby enhancing tumor cell clearance [17]. Autophagy, a highly conserved catabolic process, has been shown to play a context-dependent role in tumor progression. Depending on the glioma subtype and specific mutational profile, autophagy may function as either a tumor suppressor or promoter [18]. Inhibition of autophagy results in the accumulation of reactive oxygen species (ROS) and genomic instability, which in turn triggers endoplasmic reticulum (ER) stress and DNA damage, both of which promote tumorigenesis. Conversely, under conditions of nutrient deprivation or oxidative stress, autophagy serves as a survival mechanism by providing metabolic substrates and maintaining cellular homeostasis [19]. Recent studies have demonstrated that ampelopsin (Amp), a natural flavonoid compound, can induce both apoptosis and autophagy-dependent cell death in glioma cells. This effect is mediated through ROS generation and subsequent activation of the c-Jun N-terminal kinase (JNK) signaling pathway [20].

Beyond the four principal RCD pathways discussed above, two additional forms, panoptosis and cuproptosis, have recently been identified. PANoptosis integrates elements of pyroptosis, apoptosis, and necroptosis into a coordinated inflammatory cell death program, regulating immune cell activation and tissue homeostasis [21]. Cuproptosis is a copper-dependent process characterized by the accumulation of lipoylated mitochondrial enzymes, leading to mitochondrial dysfunction and oxidative stress [22]. Although the specific roles of panoptosis and cuproptosis in glioma remain largely unexplored, their identification provides valuable context for understanding potential adjunct pathways that could interact with traditional RCD mechanisms. Acknowledging these emerging pathways lays the foundation for future mechanistic studies and therapeutic strategies targeting glioma biology.

## Crosstalk Among Different RCD Signaling Pathways and Their Implications In Glioma

**Fig. 3:**
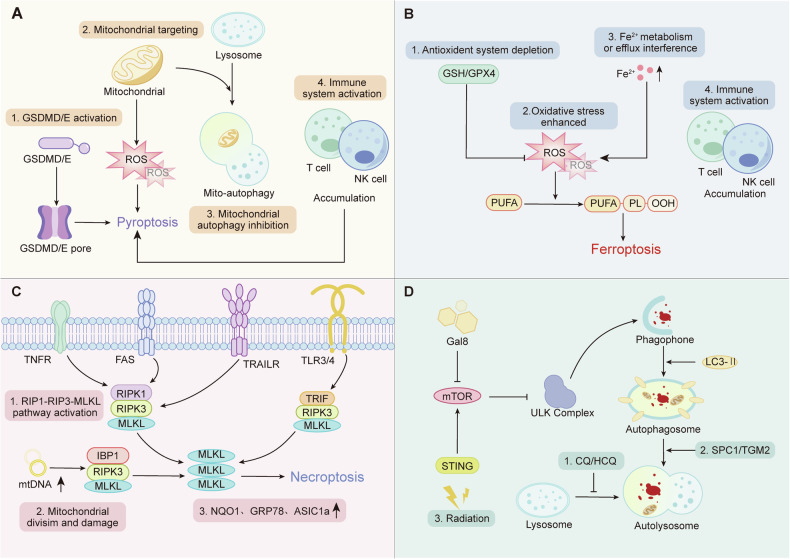
The regulatory role of pyroptosis, ferroptosis, necroptosis, and autophagy in the biological progression of glioblastoma. **A** Pyroptosis-based therapies induce caspase-mediated GSDMD/E activation, mitochondrial ROS generation, and immune cell recruitment. Strategies include photothermal systems, rAAV-mediated GSDM delivery, mitochondria-targeted nanoplatforms, and NIR-guided drug release, all designed to trigger pyroptosis and enhance antitumor immunity. **B** Ferroptosis induction is achieved by depleting antioxidant defenses (e.g., GPX4/GSH), enhancing lipid peroxidation, and disrupting Fe²⁺ metabolism. Diverse platforms including graphdiyne-based drug delivery, CRISPR-mediated gene silencing, bispecific antibodies, and dietary interventions have been used to sensitize GBM to ferroptosis and improve immunotherapy responses. **C** Necroptosis activation via RIPK1/RIPK3/MLKL signaling disrupts mitochondrial integrity and promotes immunogenic cell death. Natural compounds such as shikonin, chelerythrine, emodin, and celastrol activate necroptosis, while modulation of ASIC1a and NQO1 offers novel targets for resistant glioma populations. **D** Autophagy modulation is a promising strategy to overcome therapeutic resistance. mTOR/ULK-targeted inhibitors (e.g., CQ, HCQ), radiation-induced STING activation, and agents such as daurisoline modulate autophagic flux. Novel regulators like Gal8 and SDC1-TGM2 interactions further define autophagy’s dual role in glioma cell survival and death. Collectively, these approaches highlight the potential of RCD-targeted therapies to modulate the tumor microenvironment, enhance immunogenicity, and improve treatment efficacy in glioblastoma.

The regulation of RCD involves intricate, interconnected, and overlapping signaling pathways. At the molecular level, various forms of regulated cell death, such as pyroptosis, ferroptosis, necroptosis, and autophagy, play crucial roles in the development and progression of glioma (Fig. 3).

### Pyroptosis in Glioma

Pyroptosis is a pro-inflammatory form of RCD mediated by members of the GSDM protein family [23]. This RCD mechanism is characterized by cell swelling, plasma membrane pore formation, and the release of intracellular contents, leading to potent inflammatory responses [24]. Pyroptosis plays a critical role in maintaining tissue homeostasis and immune surveillance. It is initiated by the recognition of PAMPs and DAMPs by cytosolic pattern recognition receptors (PRRs) [25]. These signals activate the assembly of inflammasome complexes and inflammatory caspases, such as caspase-1, caspase-4, and caspase-5, ultimately leading to membrane pore formation, cell swelling, and rupture [26]. Subsequently, the activated caspases cleave GSDMs, particularly GSDMD and GSDME, resulting in the formation of membrane pores, cell lysis, and release of pro-inflammatory cytokines such as IL-1β, IL-18, and DAMPs, thereby amplifying immune responses [23].

Among the GSDM family members, GSDMB, GSDMD, and GSDME have emerged as potential biomarkers for differentiating glioma grades [8]. GSDMA has also been proposed as a candidate therapeutic target and an adjunct component in glioma immunotherapy [27]. Transcriptomic analyses based on data from The Cancer Genome Atlas (TCGA), using the Gene Expression Profiling Interactive Analysis (GEPIA) platform, reveal a positive correlation between the expression levels of GSDMD and GSDME and glioma grade (grades II–IV) [28]. Additionally, gliomas exhibiting high GSDMD or GSDME expression but low GSDMC expression are associated with significantly reduced overall survival [8]. Despite the potential of GSDMs as novel glioma biomarkers, their prognostic and predictive utility must be validated in the context of established molecular indicators such as *IDH1/2* mutations, *MGMT* promoter methylation, and *TERT* promoter mutations. In this regard, Zeng and colleagues developed a pyroptosis-related risk signature (PRRS) to predict survival outcomes and immunotherapeutic responsiveness in glioma. The high-PRRS group demonstrated increased sensitivity to temozolomide and showed enhanced responses to anti-PD-1 checkpoint blockade. Moreover, Bcl-2 inhibitors were identified as potential agents for use in combination with immunotherapy in high-risk patients [29]. Further studies reveal additional regulators of pyroptosis in glioma. For instance, GNB4 expression is significantly elevated in glioma cells compared to normal glial cells. Silencing of GNB4 promotes pyroptosis and suppresses glioma proliferation, migration, and invasion via activation of the cGAS-STING pathway, a novel axis with emerging diagnostic and therapeutic relevance in glioma [30]. In parallel, Cao et al. demonstrated that ICBP90 contributes to glioma progression and drug resistance by maintaining promoter methylation and suppressing the expression of the tumor suppressor gene *DKK3*, highlighting an epigenetic mechanism that modulates pyroptosis signaling and immune escape [31].

### Ferroptosis in Glioma

Ferroptosis is an iron-dependent form of RCD characterized by excessive lipid peroxidation and subsequent plasma membrane rupture. In murine GBM models, iron accumulation alone has been shown to directly induce ferroptosis [32]. This accumulation is driven by increased iron uptake through transferrin receptor (TFRC), degradation of intracellular iron storage proteins, and downregulation of the iron exporter SLC40A1 [33]. Excess intracellular iron promotes lipid peroxidation by generating ROS and activating iron-dependent enzymes such as lipoxygenases (ALOXs). Under the enzymatic regulation of ACSL4 and lysophosphatidylcholine acyltransferase 3 (LPCAT3), PUFAs are converted into phospholipid-PUFAs (PL-PUFAs), which are subsequently oxidized by ALOXs into phospholipid hydroperoxides (PL-PUFA-OOH), triggering ferroptotic cell death [34].

Endogenous antioxidant systems, including the GPX4 axis and the apoptosis-inducing factor mitochondria-associated 2 (AIFM2)-coenzyme Q10 pathway, play pivotal roles in counteracting ferroptosis by reducing lipid peroxides. Notably, Li et al. found that silencing GPX4 alone was insufficient to induce ferroptosis in GBM cells. However, activation of the NF-κB pathway in combination with GPX4 knockdown effectively triggered ferroptosis, indicating a synergistic role of NF-κB signaling in RSL3-induced ferroptosis [35]. Hypoxia represents a major barrier to effective glioma therapy and contributes to ferroptosis resistance. In glioma cells, hypoxic conditions upregulate SLC7A11 and increase GSH synthesis through activation of the PI3K/AKT/HIF-1α axis, thereby attenuating sensitivity to SAS-induced ferroptosis [36]. Functional genomic screening and transcriptomic analysis have identified glutathione synthetase (GSS) as a critical regulator of ferroptosis-associated radioresistance. Elevated GSS levels are strongly correlated with poor prognosis and tumor recurrence [37]. Proline-rich protein 11 (PRR11) has been found to stabilize dihydroorotate dehydrogenase (DHODH) by inhibiting its polyubiquitination and proteasomal degradation. Mechanistically, PRR11 prevents the recruitment of the E3 ubiquitin ligase HERC4 to DHODH at lysine 306 (K306), thereby promoting DHODH protein stability and ferroptosis resistance in glioma both in vitro and in vivo [38].

The molecular subtype of glioma significantly affects ferroptosis sensitivity. In IDH-mutant gliomas, disrupted lipid and redox homeostasis increases vulnerability to ferroptosis due to impaired NADPH production and glutathione metabolism [39, 40]. In contrast, EGFRvIII-expressing gliomas may exhibit ferroptosis resistance via enhanced antioxidant defenses and lipid remodeling, though these mechanisms remain underexplored [41]. Lipidomic profiling has further shown that tumors enriched in polyunsaturated fatty acids (PUFAs) are more susceptible to ferroptosis, highlighting a potential metabolic vulnerability for subtype-specific targeting [42]. Additionally, apolipoprotein C1 (APOC1) inhibits ferroptosis by suppressing Kelch-like ECH-associated protein 1 (KEAP1), facilitating Nrf2 nuclear translocation, and upregulating its downstream antioxidant targets, including heme oxygenase-1 (HO-1) and NAD(P)H:quinone oxidoreductase 1 (NQO1). APOC1 also enhances the transsulfuration pathway via upregulation of cystathionine β-synthase (CBS), thereby increasing GSH synthesis and GPX4 levels, leading to ferroptosis resistance [43].

Targeting ferroptosis has shown potential to reshape the immunosuppressive tumor microenvironment and improve responses to immune checkpoint blockade (ICB) therapies [44]. Although the cytotoxic role of ferroptosis in tumor cells is well documented, its immunological impact remains underexplored. For instance, complement C5a receptor 1 (C5aR1) prevents ferroptosis in GBM cells by enhancing methyltransferase-like 3 (MettL3)-dependent GPX4 expression through the ERK1/2 signaling pathway [45]. Neutrophil-mediated tumor cell killing has been shown to involve ferroptosis, facilitated by the transfer of neutrophil-specific granules containing myeloperoxidase (MPO) into tumor cells. This cytotoxic process is impaired by GPX4 overexpression or ACSL4 deletion, which reduces ferroptosis, necrosis, and tumor invasiveness. Clinical analysis of GBM samples supports a correlation between neutrophil infiltration, ferroptosis, and necrosis, which collectively predict poorer patient survival [46]. In a novel mechanism identified by Lu et al., glioma cells were shown to engulf neutrophils, which subsequently release MPO-containing granules intracellularly. This process is mediated by integrin-dependent adhesion and is further facilitated by LC3-associated phagocytosis (LAP), representing an immune-related route of ferroptosis induction [47].

### Necroptosis in glioma

Research on necroptosis in gliomas remains limited. Necroptosis is a distinct form of RCD that shares some morphological features with apoptosis—such as organelle swelling and membrane rupture—but differs fundamentally in its molecular mechanism, typically occurring when apoptotic pathways are suppressed [48]. Necroptosis can be triggered by activation of death receptors, such as tumor necrosis factor receptor 1 (TNFR1), or pattern recognition receptors, such as toll-like receptor 3 (TLR3). Upon stimulation, TNFR1 recruits receptor-interacting protein kinase 1 (RIPK1) via its intracellular death domain. Cellular inhibitor of apoptosis proteins (cIAPs) ubiquitinate RIPK1, which in turn activates nuclear factor κB (NF-κB) and mitogen-activated protein kinase (MAPK) signaling, promoting cell survival [49]. When RIPK1 is deubiquitinated, the membrane-bound signaling complex (complex I) transitions into complex II, leading to the activation of caspase-8 (CASP8) and the initiation of apoptosis. However, in the absence or inhibition of CASP8, RIPK1 interacts with RIPK3 to form the necrosome complex. RIPK3 subsequently phosphorylates mixed lineage kinase domain-like protein (MLKL), which oligomerizes and translocates to the plasma membrane, disrupting membrane integrity and resulting in the release of DAMPs [50]. Zhou et al. reported that necroptosis-related genes, including *MLKL*, are significantly upregulated in gliomas [51]. RIPK1 is frequently overexpressed in GBM and has been associated with unfavorable patient prognosis [52], while inhibition of RIPK3 promotes tumor growth and metastasis [53]. In general, necroptosis acts as a tumor-suppressive mechanism [54]. Approximately two-thirds of tumor cell lines evade necroptosis by downregulating RIPK3 expression, contributing to resistance against chemotherapy and radiotherapy [55, 56]. In gliomas, further suppression of necroptosis is achieved through the upregulation of cysteine and glycine-rich protein 2 (CSRP2), which activates the Janus kinase/signal transducer and activator of transcription 1 (JAK-STAT1) signaling pathway [57]. These findings suggest that restoring necroptosis in glioma cells could help overcome treatment resistance and improve therapeutic outcomes.

### Autophagy in glioma

Autophagy is an evolutionarily conserved metabolic process responsible for maintaining intracellular homeostasis. It involves the degradation and recycling of damaged proteins, defective organelles, and other cellular components through the formation of double-membrane autophagosomes [58]. Autophagy initiation is regulated by the unc-51-like kinase (ULK) complex [59]. Upon inhibition of mechanistic target of rapamycin complex 1 (mTORC1) or activation of AMP-activated protein kinase (AMPK), the vacuolar protein sorting 34 (VPS34) complex generates phosphatidylinositol 3-phosphate (PI3P), which recruits autophagy-related proteins and facilitates phagophore formation [60, 61]. During autophagosome formation, microtubule-associated protein 1 light chain 3 (LC3) is cleaved and lipidated into LC3-II, which integrates into the phagophore membrane and serves as a docking site for cargo recruitment via ubiquitin-binding adaptors. Increased LC3-II levels indicate autophagy initiation. The autophagosome then matures and fuses with lysosomes, where cargo is degraded by hydrolytic enzymes and recycled into basic nutrients [33].

Autophagy plays a dual role in gliomagenesis and therapeutic response. Under stress conditions, it promotes tumor cell survival by degrading damaged mitochondria and misfolded proteins, thereby enhancing resistance to radiotherapy and chemotherapy [62]. Proteins such as syndecan-1 (SDC1) and transglutaminase 2 (TGM2) facilitate autophagosome–lysosome fusion and enhance radiosensitivity in GBM cells [62]. Conversely, inhibition of fusion events via mucolipin 1 (MCOLN1) suppresses glioma cell proliferation and migration [63]. Glioma cells also exploit lipophagy to sustain cholesterol metabolism. In this process, sterol regulatory element-binding protein 1 (SREBP-1) upregulates lipophagy-associated genes, maintaining cholesterol homeostasis and supporting tumor growth [64]. However, excessive or dysregulated autophagy may lead to cell death. Several anticancer agents induce autophagy-dependent cytotoxicity. For example, cannabidiol (CBD) induces autophagic cell death in glioma cells by activating transient receptor potential vanilloid 4 (TRPV4) [65]. Additionally, Meyer et al. reported that the combination of pimozide and loperamide synergistically induces autophagy overactivation, disrupts lipid balance, and promotes lysosomal membrane permeabilization, ultimately resulting in glioma cell death [66].

## Targeting RCD In The Treatment of Glioma

Considering the unique mechanisms of regulated cell death pathways and their crucial involvement in glioma, targeting these processes has become a vital therapeutic strategy. The following sections offer an in-depth analysis of key targets and corresponding pharmacological agents (Table 1).

**Table 1:** Overview of potential targets of RCD in glioma therapy, associated therapeutic agents, and their clinical applications.

RCD type	Strategy	Targets	Biological effects	References
Pyroptosis	IASNDS (Salmonella + GSDMD exosomes)	GSDMD, BASP1, L-arabinose promoter	Induces pyroptosis in GBM cells, promotes immune infiltration (HMGB1, ATP, cytokines), reduces tumor invasion, and prevents postoperative recurrence	[69]
	rAAV-GSDMDNT	GSDMD, Caspase-1	Induces pyroptosis, enhances antitumor immunity, suppresses glioma growth in C6-luc rat model, increases T-cell infiltration	[71]
	Triterpene NPs (OA-iRGD + LND)	TOM70, mitochondria, pyroptosis	Induces pyroptosis in GBM cells, enhances mitochondrial targeting, prolongs survival in tumor-bearing mice, improves efficacy with lonidamine co-delivery	[72]
	M@BPTLD (BP + LND, mitophagy blocker)	Mitochondria, GSDMD, lysosome	Targets GBM mitochondria, induces pyroptosis, blocks mitophagy to enhance immune activation and dendritic cell maturation, improves immunogenic response	[73]
	PA1094T (A1094 + TMZ + anti-CD47)	CD47, apoptosis, pyroptosis	Induces apoptosis and pyroptosis, promotes macrophage repolarization to M1, enhances T-cell activation and phagocytosis, improves GBM microenvironment	[74]
	MTAB-GNRs + NIR	GSDMD, Caspase-1, NLRP3	Induces pyroptosis in 3D glioma tumoroids (GL261, U-87 MG); avoids classical apoptosis; confirms photothermal-triggered regulated cell death	[75]
Ferroptosis	ABX + TMZ	XPC, ERCC1, HOXM1, GPX4	Synergistically enhances TMZ sensitivity in GBM cells, induces sustained DNA damage, activates ferroptosis pathway, significantly improves efficacy in PDO models and prolongs survival in mouse models	[76]
	IGF2BP3 knockdown	GPX4 via m6A/IGF2BP3	Suppresses glioma cell growth, enhances ferroptosis by destabilizing GPX4 mRNA, increases microglial phagocytosis, prevents tumor formation in xenograft models	[78]
	GFR (GDY + FIN56 + RAP) + PTT	GPX4, BBB, pH/heat-triggered release	Induces ferroptosis via GPX4 suppression, enhances FIN56 release under acidic/heat conditions, inhibits tumor growth, prolongs survival in GBM mouse model	[80]
	CRISPR-Cas9 EVs targeting GSS + RT	GSS, GPX4, GSH synthesis	GSS depletion disrupts GSH synthesis, inactivates GPX4, enhances ferroptosis with radiation, improves therapeutic response; BBB-penetrating CRISPR delivery achieves 67.2% editing in GBM tissue	[37]
	S-biAb/dEGCG@NPs	B7-H3, CD3, IFN-γ pathway	Enhances ferroptosis and immune activation in GBM, improves intracranial delivery and survival, boosts ICB efficacy with targeted nanoparticle delivery	[81]
	GPR68 inhibition (shRNA/OGM)	GPR68, lipid peroxidation	Induces ferroptosis in GBM cells, reduces tumor cell viability in zebrafish xenograft models, low off-target toxicity; validates GPR68 as a therapeutic target	[82]
	Brucine	ATF3, NOX4, SOD1, catalase, xCT	Induces ferroptosis in glioma via ATF3-mediated H2O2 and iron accumulation, triggers lipid peroxidation; effects reversed by ferroptosis inhibitors or ATF3 knockdown	[84]
	Erianin	REST, LRSAM1, SLC40A1	Induces ferroptosis in TMZ-resistant glioma stem cells by degrading SLC40A1 via REST-LRSAM1 axis; enhances TMZ sensitivity and suppresses tumor phenotype	[85]
	Ginsenoside Rg5	NR3C1, HSPB1, NCOA4	Activates ferroptosis in glioma stem cells via NR3C1-mediated regulation of HSPB1 and NCOA4; inhibits GSC viability, invasion, self-renewal, and tumor progression in vivo	[86]
Necroptosis	Shikonin	PSMB8/9/10, PSME1/2/3, SOX2, CD44, CHI3L1, CD24	Induces necroptosis and inhibits glioma stemness by suppressing proteasome activity and stem cell markers (SOX2, CD44, CHI3L1, CD24)	[89]
	Chelerythrine (CHE)	RIP1, RIP3, mtROS, Drp1, PINK1, Parkin, Ca2+ influx	Induces necroptosis via mtROS-driven RIP1/RIP3/Drp1 signaling and mitochondrial dysfunction; mitophagy limits necroptosis; Ca2+ influx acts as priming signal	[90]
	Emodin	TNF-α, RIP1, RIP3, MLKL	Induces necroptosis via TNF-α/RIP1/RIP3/MLKL pathway in glioma cells; effect suppressed by Nec-1 and GSK872; validated in vitro and in vivo	[91]
	Celastrol derivative (compound 6i)	RIP1, RIP3, MLKL	Enhances anti-glioma activity via RIP1/RIP3/MLKL-mediated necroptosis; more potent than celastrol; effective in vitro and in zebrafish model with BBB permeability	[92]
	PBI-05204 (oleandrin-containing extract)	RIPK1, RIPK3, GRP78, CD44, NANOG	Induces necroptosis and suppresses stemness in GBM stem cells via RIPK1/RIPK3 activation and GRP78 inhibition; reduces tumorigenesis and spheroid formation	[93]
	2-Methoxy-6-acetyl-7-methyljuglone (MAM)	NQO1, O2-, Ca2 + , JNK1/2	Induces necroptosis in GBM via NQO1 activation and O2-/Ca2 + /JNK1/2 signaling; reduces tumor growth in zebrafish model	[94]
	ASIC1a activation (e.g., by acidosis or MitTx1)	ASIC1a, RIPK1, MLKL (partially), necroptosis-like cascade	Prolonged ASIC1a activation induces necrosis in GSCs via RIPK1-dependent but MLKL-independent pathway; reduces tumorsphere formation in acidic microenvironment	[95]
Autophagy	Hydroxychloroquine (HCQ) + RT + TMZ	Autophagic vacuoles (AVs), LC3-II	Autophagy inhibition achieved at 600 mg/day HCQ; higher doses caused severe toxicity; inconsistent autophagy inhibition; no significant survival improvement observed	[98]
	Galectin-8 targeting (genetic suppression)	Galectin-8, mTORC1, TFEB, Ragulator-Rag complex	Hypoxia-induced Gal-8 enhances autophagy to sustain GSC stemness via mTORC1 inactivation and TFEB activation; Gal-8 suppression inhibits tumor growth and improves survival in mice	[100]
	Targeting SDC1/TGM2/FLOT1/BHMT complex	SDC1, TGM2, FLOT1, BHMT, autophagosomes/ lysosomes	SDC1-TGM2-FLOT1-BHMT complex maintains autophagic flux in irradiated GBM cells by promoting autophagosome-lysosome fusion, enhancing radioresistance	[101]
	IRAK1 knockdown or STING inhibition	IRAK1, FOXA2, PRDX1, HECTD3, autophagic cell death	IRAK1 promotes radioresistance via stabilizing PRDX1, suppressing autophagic death; STING-FOXA2-IRAK1 axis regulates PRDX1 levels; knockdown sensitizes glioma to radiotherapy	[102]
	Daurisoline (DAS) ± Temozolomide (TMZ)	PI3K/AKT/mTOR, autophagic flux, caspase-3/6/9, EMT markers	DAS impairs late-stage autophagic flux via PI3K/AKT/mTOR, induces apoptosis, inhibits EMT, and enhances TMZ chemosensitivity in vitro and in vivo	[103]

### Pyroptosis-based Therapeutic Strategies

In cancer treatment, pyroptosis has garnered significant attention as a mechanism for eliminating tumor cells while simultaneously stimulating antitumor immune responses [67]. Therapeutically inducing pyroptosis, particularly in combination with immunotherapy, has emerged as a promising strategy for treating GBM.

Chen et al. developed a photothermal therapy (PTT) system using polymeric semiconductor-based nanoparticles with high optical stability, modified with mesoporous silica for improved biocompatibility [68]. These nanoparticles were functionalized with polyethylene glycol (PEG) and cyclic RGD (cRGD) peptides to enhance tumor targeting and effectively induced pyroptosis in vitro, demonstrating potential for GBM therapy with reduced off-target effects. Zhang et al. engineered an injectable bacteria-hydrogel superstructure designed to target peritumoral GBM satellites. This system triggered pyroptosis in GBM cells and activated both innate and adaptive immunity, preventing postoperative recurrence in a murine model [69]. Similarly, Song et al. identified lomitapide (LMP) as a pyroptosis-inducing agent via high-throughput screening. They further developed a biomimetic nanoparticle platform (RFA NPs) with excellent BBB penetration and tumor-targeting efficiency to enhance delivery [70]. Yuan et al. used a recombinant adeno-associated virus (rAAV) expressing GSDM to transiently disrupt the BBB and recruit tumor-infiltrating lymphocytes, thereby inducing pyroptosis. This effect was amplified by combination with programmed death-ligand 1 (PD-L1) blockade, improving survival outcomes [71].

To address the issue of mitophagy-mediated immune suppression, Gao et al. designed tertiary amine-modified triterpenoid nanoparticles that targeted mitochondria to induce pyroptosis, significantly extending survival in GBM-bearing mice [72]. Ye and colleagues developed a mitochondria-targeting pyroptosis system by modifying lonidamine (LND) onto black phosphorus (BP) nanosheets encapsulated in macrophage membranes, enabling BBB penetration and enhancing pyroptosis by inhibiting mitophagy and promoting dendritic cell maturation under near-infrared (NIR) irradiation [73]. Additional platforms, including second near-infrared (NIR-II) photoacoustic imaging (PAI)-guided nanoprodrugs that combine A1094 dye, temozolomide (TMZ), and anti-CD47 antibodies, have been shown to simultaneously trigger pyroptosis and enhance T cell responses in GBM [74]. Photothermal strategies using gold nanorods modified with (16-mercaptohexadecyl) trimethylammonium bromide were also developed to precisely regulate pyroptosis via laser intensity control [75].

### Ferroptosis-based Therapeutic Strategies

Ferroptosis represents an emerging therapeutic modality in glioma due to its ability to overcome resistance to radiotherapy and chemotherapy. Strategies to induce ferroptosis in glioma generally follow two main approaches.

First, by inhibiting the endogenous antioxidant systems that protect glioma cells from oxidative stress. Zhai et al. showed that albumin-bound paclitaxel (ABX) combined with TMZ enhanced ferroptosis via modulation of *HOXM1* and *GPX4* expression [76]. Similarly, suppression of the Fanconi anemia gene *FANCD2* increased Fe²⁺ accumulation through upregulation of DMT1 and downregulation of GPX4, thereby inducing ferroptosis in SHH-medulloblastoma cells [77]. Insulin-like growth factor 2 mRNA-binding protein 3 (IGF2BP3), an m⁶A reader protein, was shown to regulate ferroptosis by binding to *GPX4* mRNA and modulating its translation [78, 79]. Graphdiyne (GDY) has been explored as a delivery platform for the ferroptosis inducer FIN56. Under 808 nm irradiation, GDY enabled pH-responsive drug release and enhanced ferroptotic efficacy in GBM cells [80].

Second, activating ferroptosis-related pathways using innovative delivery or gene-editing technologies. Liu et al. constructed a non-viral extracellular vesicle system (Ang/TAT-EVs carrying Cas9/sgRNA), which efficiently knocked down *GSS* in GBM cells, thereby enhancing ferroptosis sensitivity [37]. Fan et al. designed a bispecific anti-B7-H3×CD3 antibody combined with matrix metalloproteinase-2 (MMP-2)-responsive nanoparticles (S-biAb/dEGCG@NPs) to simultaneously trigger ferroptosis and boost immunotherapy efficacy [81]. OGM, a small molecule targeting GPR68, was also reported to promote ferroptosis in vivo [82]. Another GDY-based nanoplatform (GDY-FIN56-RAP) was developed to suppress *MS4A4A* expression in TAMs, enhancing M1 polarization and sensitizing tumors to anti-PD-1 therapy [83].

Natural compounds such as brucine, erianin, and ginsenoside Rg5 have also demonstrated ferroptosis-inducing effects. Brucine induces ferroptosis via ER stress–mediated H₂O₂ accumulation [84]; erianin inhibits REST-mediated transcriptional repression, leading to SLC40A1 degradation via *LRSAM1* and ferroptosis in TMZ-resistant glioma stem cells [85]; and ginsenoside Rg5 promotes ferroptosis through the NR3C1 pathway [86]. Upadhyayula et al. proposed that dietary cysteine and methionine deprivation (CMD) could synergize with GPX4 inhibitors such as RSL3 to enhance ferroptosis and lipid peroxidation in glioma models and patient-derived organoids [87].

### Necroptosis-based Therapeutic Strategies

Recent research has underscored necroptosis, a caspase-independent RCD pathway controlled by the RIPK1/RIPK3/MLKL signaling axis, as an effective mechanism for eliminating glioma cells, particularly those resistant to conventional treatments such as chemotherapy and radiotherapy. The induction of necroptosis disrupts tumor survival pathways and offers novel avenues for therapeutic intervention.

Several natural compounds have been identified as necroptosis inducers in glioma. For instance, shikonin induces reactive oxygen species (ROS) production and activates the RIPK1/RIPK3/MLKL pathway to trigger necroptosis in glioma cells [88, 89]. Chelerythrine (CHE) promotes mitochondrial ROS generation, depolarization, ATP depletion, and fragmentation. It forms an mtROS-mediated RIPK1–RIPK3–Drp1 complex that enhances Drp1 translocation to mitochondria, thus triggering necroptosis [90]. Furthermore, emerging evidence suggests that emodin induces necroptosis by activating the TNF-α/RIPK1/RIPK3 signaling cascade [91]. Similarly, celastrol, a quinone methide triterpenoid, activates the RIPK1/RIPK3/MLKL axis and suppresses U251 glioma cell proliferation in zebrafish xenograft models [92]. Oleandrin, a cardiac glycoside and the active ingredient in PBI-05204, targets glioblastoma stem cells by downregulating glucose-regulated protein 78 (GRP78) and inducing necroptotic cell death [93]. Moreover, NQO1, frequently overexpressed in GBM and correlated with tumor malignancy, is directly activated by 2-methoxy-6-acetyl-7-methyljuglone (MAM), leading to necroptosis [94]. In addition, mild acidosis (pH 6.6) has been shown to activate acid-sensing ion channel 1a (ASIC1a), inducing necroptosis in glioblastoma stem cells [95]. These findings suggest that necroptosis-based therapies—particularly those utilizing natural compounds or exploiting stress-responsive pathways—may enhance immunogenic cell death and offer novel combinatory strategies in glioma immunotherapy.

### Targeting Autophagy

Autophagy plays a critical role in glioma progression and treatment response, particularly in promoting tumor cell survival following radiotherapy, which contributes to radioresistance and malignant recurrence [96].

Pharmacologic inhibition of autophagy using agents such as chloroquine (CQ) and hydroxychloroquine (HCQ) has been shown to sensitize tumor cells to radiation [97]. A phase I/II clinical trial combining HCQ with radiotherapy (RT) demonstrated improved overall survival (OS) in GBM patients, although additional HCQ treatment did not significantly extend OS in some cohorts [98]. Another phase IB trial confirmed the feasibility of combining CQ with concurrent RT and TMZ [99]. Recent studies have identified several autophagy-associated factors implicated in glioma resistance to radiotherapy and chemotherapy. Liu et al. reported that glioma stem cells overexpress galectin-8 (Gal-8), which binds to the Ragulator–Rag complex, suppresses mTORC1 signaling, and initiates autophagy [100]. Zeng et al. found that syndecan-1 (SDC1, CD138) interacts with transglutaminase 2 (TGM2) in radioresistant GBM cells, promoting autophagosome–lysosome fusion to mitigate radiation-induced stress [101]. Radiotherapy has also been shown to upregulate interleukin-1 receptor-associated kinase 1 (IRAK1) via activation of stimulator of interferon genes (STING) signaling. Paradoxically, this suppresses autophagy and contributes to secondary radiation resistance in glioma [102]. Therapeutically, daurisoline was shown to inhibit TMZ-induced autophagy by activating the PI3K/AKT/mTOR pathway, thus reducing glioma proliferation and enhancing chemosensitivity [103]. Collectively, these findings support the potential of autophagy-targeting therapies to overcome glioma resistance and improve treatment outcomes.

## Clinical Translation of RCD-Targeted Strategies In Glioma

Despite extensive preclinical evidence supporting the therapeutic relevance of RCD in glioma, clinical translation remains in its early stages. Among the RCD subtypes, autophagy has been most frequently investigated in early-phase clinical trials, primarily through the use of HCQ or CQ in combination with radiotherapy and TMZ. Trials such as NCT05009992 and NCT02432417 have demonstrated acceptable safety profiles, although effective autophagy inhibition was inconsistently achieved, and no survival benefit has been conclusively demonstrated.

Pyroptosis has been indirectly explored through oncolytic virotherapy—for example, herpes simplex virus (HSV) G207 in trial NCT02197169—which induces innate immune responses and pro-inflammatory cytokines such as IL-1β. In contrast, therapies targeting ferroptosis and necroptosis remain largely preclinical, with promising findings emerging from studies involving cold atmospheric plasma, fatty acid metabolism modulation, and immune-enhancing compounds in xenograft and organoid models. A summary of completed clinical trials targeting RCD pathways in glioma is presented in Table 2.

**Table 2:** Clinical advancements in targeting RCD pathways in glioma.

Trial ID	Phase	RCD target	Design	Population	Final points	Intervention/Strategy	Key clinical findings
NCT02432417	I/II	Autophagy	Radiotherapy + chemo ± CQ/HCQ	Newly diagnosed GBM	MTD, tolerability, PFS	HCQ + RT + TMZ	MTD 600 mg/day HCQ. No clear survival benefit yet
NCT02378532	I	Autophagy	CQ as radiosensitive	GBM (EGFRvIII + )	Safety, radiosensitization effect	CQ + RT + TMZ	Studied radiosensitization via autophagy inhibition
NCT00486603	I/II	Autophagy	CQ with chemo-radiotherapy	Newly diagnosed GBM	Feasibility, tolerability	HCQ + RT + TMZ	HCQ well tolerated; feasible inhibition with standard therapy
NCT01894633	I	Autophagy	CQ with standard therapy	Newly diagnosed GBM	Early-phase dose	CQ + RT + TMZ	Safe, potential radiosensitizing effect
NCT02619864	I	Autophagy	Dendritic vaccine + TMZ	GBM	Immune activation markers	Dendritic Cell Vaccine + TMZ	Autophagy-induced immune modulation noted
NCT00805376	I/II	Autophagy via OV	Oncolytic virus-based	GBM	Proteomic changes, safety	Oncolytic virus inducing autophagy	Autophagic remodeling seen; well tolerated

Although hydroxychloroquine-based combinations have demonstrated acceptable safety profiles in early-phase studies, including NCT02432417 and NCT00486603, none have shown consistent or significant survival benefits in randomized or non-randomized settings. This highlights the importance of transparently reporting negative or neutral findings to avoid publication bias and to guide realistic clinical expectations.

Overall, while RCD-targeted therapies offer a novel strategy to overcome glioma resistance and recurrence, no late-phase clinical trials have yet validated their clinical efficacy. Future progress will require the development of selective and potent RCD modulators, biomarker-guided patient stratification, and rational combination with existing standard-of-care therapies.

## Challenges in Targeting RCD Pathways in Glioma

RCD plays a critical role in the initiation, progression, and therapeutic response of gliomas. As our understanding of the molecular mechanisms underlying various RCD pathways deepens, novel strategies targeting pyroptosis, ferroptosis, necroptosis, and autophagy are emerging as promising approaches for glioma treatment. Given the highly invasive and therapy-resistant nature of gliomas, the advancement of precision medicine and personalized therapies represents both a challenge and an opportunity. Progress in genomics and multi-omics technologies is enabling tailored interventions based on individual molecular profiles, including tumor-specific mutations, epigenetic modifications, and tumor microenvironment characteristics.

Nevertheless, several obstacles hinder the clinical application of RCD-based therapies. Tumor heterogeneity leads to patient-specific variability in RCD pathway activation, highlighting the need for reliable molecular biomarkers to guide treatment decisions. The limited permeability of the BBB restricts drug delivery, necessitating the development of advanced delivery platforms, particularly nanotechnology-based systems. Additionally, the complexity of the glioma immune microenvironment introduces variability in responses to combined RCD-immunotherapy approaches. Future research should focus on biomarker discovery, drug delivery optimization, and integration of immunotherapy to translate RCD-based strategies into clinically effective, individualized glioma treatments.

## Conclusion and Perspectives

Gliomas are the most common primary brain tumors and are defined by their aggressive invasion, cellular heterogeneity, and high resistance to conventional treatments. These features contribute to poor prognosis and frequent tumor recurrence. RCD plays a multifaceted role in glioma biology, acting as both a tumor-suppressive and tumor-promoting mechanism. Pyroptosis, ferroptosis, necroptosis, and autophagy are tightly regulated and intricately linked to the tumor immune microenvironment.

Therapeutically targeting these RCD pathways offers a promising strategy for overcoming treatment resistance and improving immunotherapy efficacy. The integration of precision medicine approaches, including multi-omics profiling and nanotechnology-based drug delivery systems, is expanding the potential for personalized glioma therapy. In particular, for instance, the combination of ferroptosis inducers with immune checkpoint inhibitors has the potential to enhance antitumor immune responses while simultaneously impairing tumor survival. Such multimodal, synergistic interventions may improve therapeutic outcomes and reduce adverse effects associated with conventional therapies. Future investigations should also consider rational combination strategies to enhance therapeutic efficacy. These may include integrating ferroptosis inducers with immune checkpoint blockade, pairing oncolytic virotherapy with phagocytosis-enhancing agents such as CD47 inhibitors, or incorporating autophagy inhibitors into chemoradiation regimens for selected molecular subtypes. Such approaches, guided by predictive biomarkers and pharmacodynamic readouts, may offer a more precise and synergistic framework for glioma treatment.

However, challenges such as intratumoral heterogeneity, limited BBB permeability, and immunosuppressive signaling must be addressed. Future research should aim to optimize RCD-targeted therapies, clarify their immunological impact, and advance their integration into multimodal treatment regimens, ultimately improving outcomes for glioma patients.
